# Multiple Myeloma: A Rare Presentation As Unilateral Pleural Effusion

**DOI:** 10.7759/cureus.54789

**Published:** 2024-02-23

**Authors:** Kalyani Deshmukh, Kajal Hatgoankar, Milind Pande, Parag Sabale, Nandkishor J Bankar

**Affiliations:** 1 Pathology, Datta Meghe Medical College, Datta Meghe Institute of Higher Education and Research, Nagpur, IND; 2 Anesthesia, Indira Gandhi Medical College, Maharashtra University of Health Sciences, Nagpur, IND; 3 Microbiology, Jawaharlal Nehru Medical College, Datta Meghe Institute of Higher Education and Research, Wardha, IND

**Keywords:** m band, pleural fluid immunofixation, plasma cells, myelomatous pleural effusion (mpe), multiple myeloma (mm)

## Abstract

Multiple myeloma (MM) is a hematologic malignancy characterized by the clonal proliferation of plasma cells in the bone marrow. It commonly presents with bone pain, anemia, renal failure, and hypercalcemia. Pleural effusion in MM usually has multiple causes, but it is rare for the effusion to be due to myelomatous deposition of the pleura. Here, we present a rare case in which the patient presented to the outpatient department with a dry cough, breathlessness, and generalized weakness. The patient was diagnosed with MM with myelomatous pleural effusion (MPE), highlighting the importance of considering MM as a differential diagnosis in patients with atypical presentations. MPE indicates a poor prognosis, and early consideration of MPE can lead to an earlier diagnosis and a more effective treatment of MM.

## Introduction

Multiple myeloma (MM) is a malignant plasma cell disorder that mainly affects the skeletal system and bone marrow. Pleural effusions are rare and usually occur in other conditions that coexist with MM [1]. The age group affected by MM is between 65 and 75 years old [2]. MM comprises around 10-20% of all hematological malignancies and is more common in males [3]. Myelomatous pleural effusion (MPE) is a rare complication of MM and is found in around 1% of the cases with MM. The life expectancy is four months after the diagnosis of pleural effusion [1]. Pleural effusion in MM usually has multiple causes, but it is rare for the effusion to be due to myelomatous deposition of the pleura. It often occurs in the later stages of the disease and is generally associated with an unfavorable prognosis [4]. MPE occurs in 1% of cases as a direct consequence of MM [5]. Here, we present a rare case of MPE, which presents with a dry cough, dyspnea, and generalized weakness. The diagnosis of MM with MPE was established.

## Case presentation

**Figure 1:**
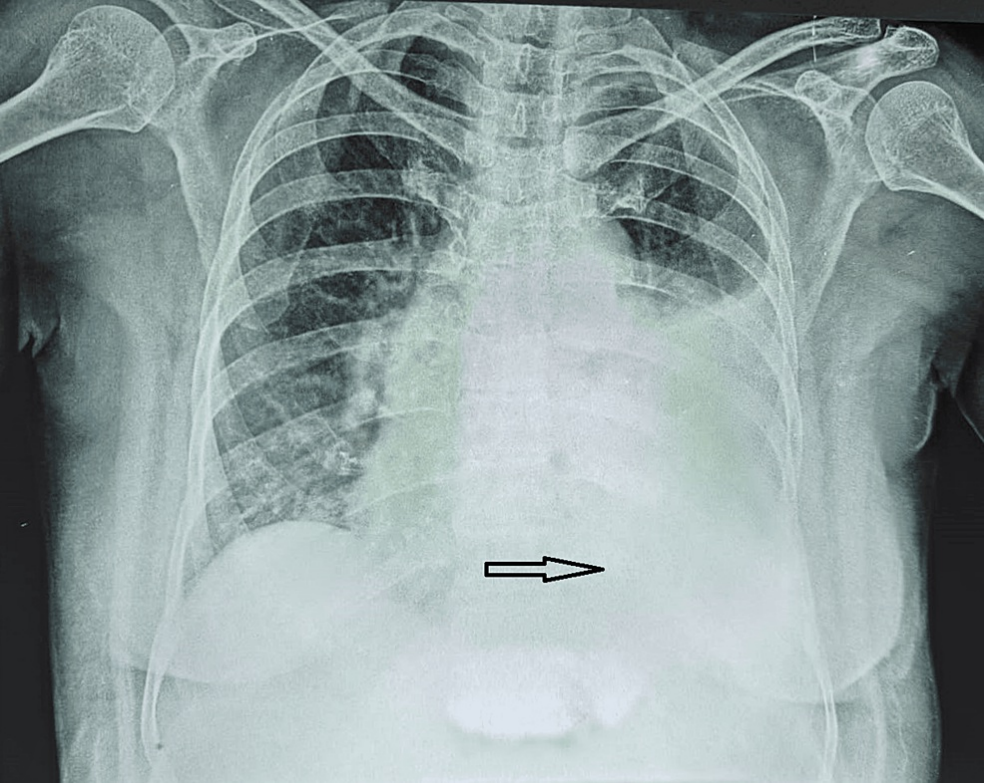
Chest radiograph shows sided pleural effusion

**Figure 2:**
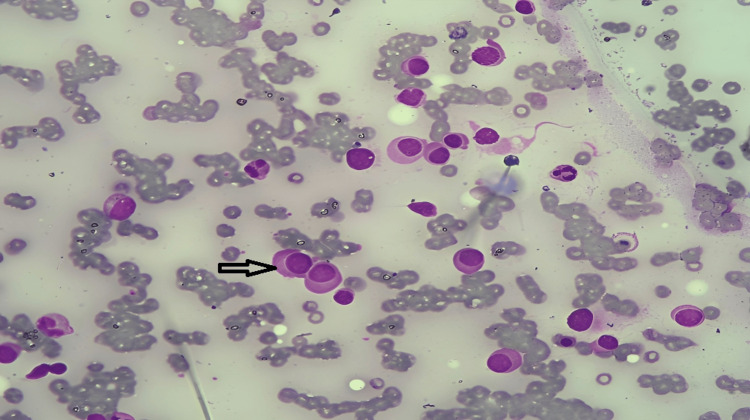
Bone marrow aspiration shows plasma cells

**Figure 3:**
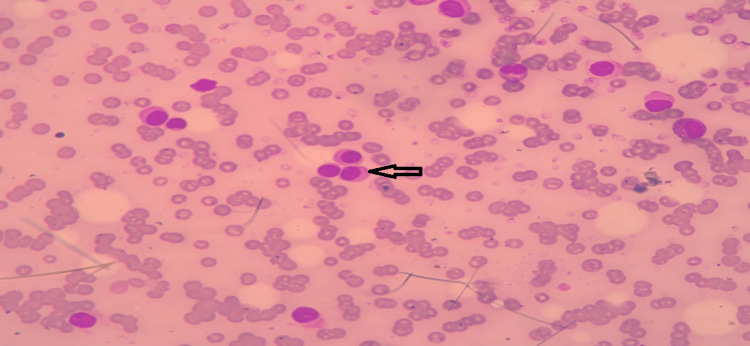
Cytology of pleural fluid shows plasma cells

**Figure 4:**
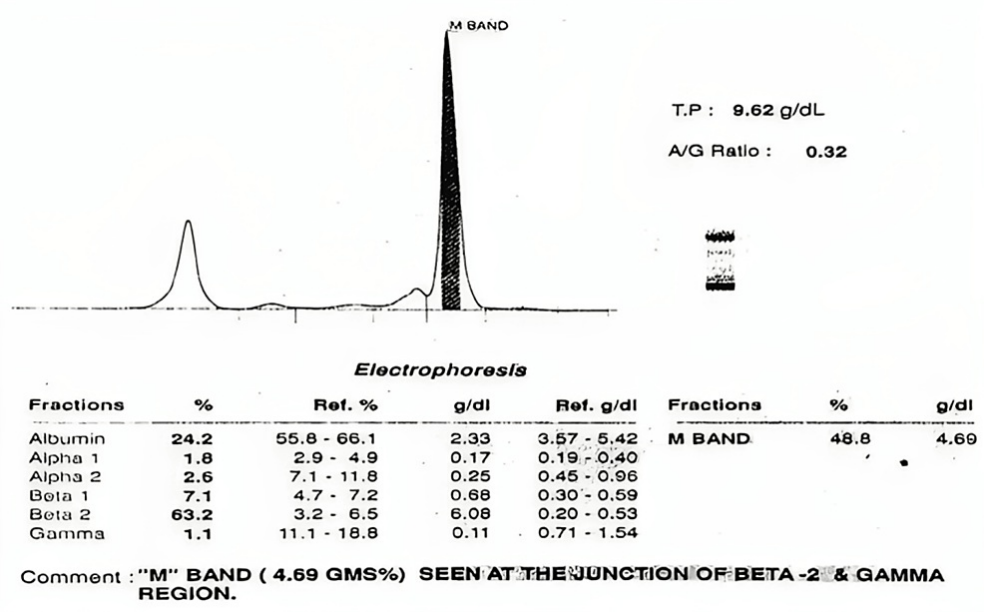
Serum protein electrophoresis shows the "M" band

A 70-year-old male presented with a dry cough, breathlessness, and generalized weakness. The patient had a history of hypertension for 15 years, no history of diabetes, and no history of past major illnesses like tuberculosis or any major surgery. On examination, severe pallor and stony dullness were present in the infraaxillary region of the left side of the chest. The chest radiograph revealed a left-sided pleural effusion (Figure 1), which was later verified by ultrasound. A peripheral blood smear revealed normocytic normochromic anemia with rouleaux formation. Examination of the bone marrow showed hypercellular marrow with suppressed erythropoiesis and myelopoiesis and 67% plasma cells, suggesting MM (Figure 2). Pleural fluid cytology revealed abnormal plasma cells (Figure 3). The beta-2 microglobulin level was elevated (3.24 mg/L). Serum protein electrophoresis and pleural fluid electrophoresis showed an ‘M Band’ at the junction of the beta-2 and gamma regions (Figure 4). A skull radiograph revealed several osteolytic lesions. The total leucocyte count of the pleural fluid was 6825/mm3 with 30% neutrophils and 70% lymphocytes; the pleural fluid protein was 9 g/dL; and the glucose was 39 mg/dL; the lactate dehydrogenase of the pleural fluid was 4800 IU/L; the Ziehl-Neelsen stain of the pleural fluid was negative for acid-fast bacilli; and culture sensitivity did not show any growth of the organism. Serum and pleural fluid immunofixation revealed an immunoglobulin G lambda light chain band. The patient died within six months after the diagnosis.

Routine investigations are shown in Table 1.

**Table 1:** Routine blood investigation of the patient

Parameter	Results (unit)	Reference range (unit)
Hemoglobin	5.4 (gram/deciliter)	14-18 (gram/deciliter)
Serum creatinine	2.97 (milligram/deciliter)	0.57–1.11 (milligram/deciliter)
Serum urea	63 (milligram/deciliter)	15-39 (milligram/deciliter)
Serum uric acid	11.6 (milligram/deciliter)	4-8.5 (milligram/deciliter)
Serum calcium	11 (milligram/deciliter)	8.5–10 (milligram/deciliter)
Total proteins	9.62 (gram/deciliter)	6-8 (gram/deciliter)
Serum albumin	2.3 (gram/deciliter)	3.5–5.0 (gram/deciliter)
Serum globulin	6 (gram/deciliter)	2.0-3.5 (gram/deciliter)

## Discussion

M-proteins in the serum, lytic bone lesions, and renal insufficiency are the characteristics of MM. Regrettably, MM is a terminal condition. This rare form of malignancy constitutes around 1% of all cancers and accounts for 10-20% of hematologic malignancies. MM is most commonly diagnosed in people aged 50 to 75 years, with an average age of 65 years. Evidence of bone marrow plasmacytosis (>10%), lytic bone lesions, and the M component in the serum and/or urine are the defining features of MM. 97% of patients with myeloma have intact immunoglobulin or a free light chain that can be identified in blood or urine by protein electrophoresis, immunoelectrophoresis, or immunofixation [6]. The acronym CRAB is used to summarize the key features of MM, which include "hypercalcemia, renal failure, anemia, and lytic bone lesions," especially in the skull. The disease has a gradual onset, and in the early stages, patients may experience weakness and weight loss. As the disease progresses, symptoms related to skeletal lesions, kidney disease, bone marrow failure, and hyperviscosity become more prominent. Bone pain in the back and ribs is the most common symptom of MM, which is worsened by movement and is caused by osteolytic lesions and osteosclerosis.

In our case, the presentation with dry cough, breathlessness, and generalized weakness is an unusual way for MM to manifest, as the disease typically affects only the bone and bone marrow, with extramedullary involvement being rare. Pleural effusion is observed in fewer than 6% of cases of MM, while malignant MPE occurs in less than 1% of cases [7]. Oudart et al. provided a comprehensive overview of the six underlying factors associated with the appearance of pleural effusion in individuals with MM [8]. These factors include congestive heart failure caused by amyloidosis, chronic renal failure, renal tubule paraprotein infiltration, hypoalbuminemia, direct extension of the pleural fluid from neighboring tissues, pulmonary embolism, obstruction of lymphatic drainage, secondary neoplasms, and involvement of myeloma in the pleura. In our patient, the chest radiograph was normal except for the presence of pleural effusion. Although renal function tests showed a slight increase in serum creatinine level, no obvious signs of renal failure were present in the patient. Thus, non-myelomatous causes of pleural effusion were ruled out.

Extramedullary involvement in MM is rare, and the disease is primarily localized within the bone and bone marrow. While rib and sternum involvement is frequently observed, occurrences of pulmonary parenchymal or pleural involvement are very rare and may present as a lung mass, a pleural effusion, pulmonary nodules, a pleural nodule, or an infiltration of the tracheobronchial region [9]. Myelomatous pleural involvement can occur through several mechanisms, including the extension of an adjacent skeletal lesion, lymph node infiltration in the mediastinum with resultant lymphatic obstruction, and direct involvement of the pleura by myeloma [2]. The condition is described as MPE when malignant plasma cells are identified in the pleural fluid. In our case, the specific cause of MPE was not determined.

If only a few plasma cells appeared in the pleural fluid, it is not diagnostically significant, but the presence of a large number of plasma cells with aberrant morphology in the pleural fluid implies MM. Reactive plasmacytosis is generally accompanied by lymphocytes, neutrophilic leukocytes, and reactive mesothelial cells in tuberculosis and Hodgkin lymphoma, with rarely more than 15-20% of the cells involved [10]. Recognizing atypical plasma cells in the pleural fluid is crucial for distinguishing malignant from reactive plasma cell infiltration [11]. MPEs have a poor prognosis, as they are frequently identified in the advanced stages of the disease, and even with aggressive therapy, the median survival period is typically less than four months.

## Conclusions

MPE as an early manifestation of MM is extremely rare. The initial diagnosis of MPE is facilitated by the detection and identification of malignant plasma cells in the pleural fluid. MPE has a poor prognosis since it is frequently identified in the advanced stages of the disease, and even with aggressive therapy, the median survival period is typically less than four months. Chemotherapy with bortezomib, lenalidomide, second-generation carfilzomib, or dexamethasone remains the first line of treatment. It is crucial to take into account the potential diagnosis of MM when elderly individuals present with pleural effusion. Early consideration of MPE can lead to a more effective diagnosis and management of MM.
